# A systematic review of predictors of college students’ subjective well-being: evidence from pre- and post-pandemic literature

**DOI:** 10.3389/fpubh.2026.1793063

**Published:** 2026-04-16

**Authors:** Jianmin He, Mohd Rustam Mohd Rameli

**Affiliations:** 1 Faculty of Educational Sciences and Technology, Universiti Teknologi Malaysia, Johor Bahru, Malaysia; 2 Mental Health Centre, School of Marxism, Foshan Polytechnic, Foshan, China

**Keywords:** Chinese college students, COVID-19 pandemic, JBI critical appraisal, predictors, subjective well-being, systematic literature review

## Abstract

**Background:**

The COVID-19 pandemic significantly affected the mental health of university populations, necessitating a systematic synthesis of the predictors of subjective well-being among Chinese college students.

**Methods:**

Following PRISMA 2020 guidelines, the researchers searched PubMed, Web of Science, and Scopus for peer-reviewed studies published between 2018 and 2024, completing the final search on December 30, 2024. Methodological quality was evaluated using design-specific JBI appraisal tools to accommodate the diverse longitudinal, quasi-experimental, qualitative, and cross-sectional methodologies within the sample. The analytic process utilized a two-stage thematic synthesis involving deductive data extraction followed by inductive theme generation to maintain methodological precision.

**Results:**

The final sample included 34 studies comprising 15,301 participants and revealed six primary predictive clusters for well-being, including social support, interpersonal dynamics, physical activity, and individual resilience. Longitudinal and quasi-experimental findings indicate that familial cohesion, leisure crafting, and adaptive coping strategies are sustained predictors of happiness during the post-pandemic recovery phase. Qualitative data further elucidate subjective challenges regarding digital temperance and the construction of self-identity in virtual environments.

**Conclusion:**

This study provides an empirical framework to guide higher education administrators and policymakers in developing targeted mental health interventions tailored to evolving academic environments.

## 1 Introduction

### 1.1 Background on subjective well-being and its importance for college students

Subjective well-being plays a crucial role in the lives of college students, presenting a tapestry of emotions, life satisfaction, and a multifaceted understanding of happiness (1). This psychological concept is often viewed as an individual’s self-assessment of their own life, spanning emotional reactions and cognitive judgments (2). The primary components of subjective well-being include life satisfaction, the positive affect, and the negative affect (3).

College students, often navigating a tumultuous yet transformative phase (4), are subject to various factors that can sway their subjective well-being. Academic stress stands out as a dominant determinant influencing a student’s day-to-day experience (5–7). However, a supportive academic environment and effective coping mechanisms can help mitigate these impacts, allowing students to maintain a well-balanced and positive outlook (8). Another pivotal determinant is the social network and the quality of relationships that college students foster (9). Interpersonal relationships are a vital component of human life, and the college period is typically marked by the development of significant friendships. These connections offer emotional stability, a sense of togetherness, and a feeling of belonging, all of which enhance an individual’s subjective well-being (10).

Conversely, when individuals experience social isolation or are embroiled in conflictual relationships, they are more likely to feel lonely, which in turn can intensify their negative emotional states (11). Intrinsic motivation plays a crucial role in enhancing subjective well-being (12). When students participate in activities that resonate with their personal interests and values, they gain a profound sense of fulfillment and purpose. This alignment significantly boosts their happiness and overall satisfaction (13). Moreover, the ability to make autonomous decisions and pursue self-determined academic goals contributes to a more positive outlook on life. It also helps to mitigate the adverse effects of stressors (14).

Furthermore, personality traits significantly shape an individual’s subjective well-being. Optimism and resilience, for instance, are strongly linked to higher levels of well-being, empowering individuals to confront adversities with a positive mindset (15, 16). Conversely, neuroticism often correlates with lower well-being due to heightened anxiety and mood instability (17). Understanding these traits is crucial for developing personalized interventions to enhance well-being among college students. Beyond personality, environmental factors such as financial stability and living conditions also profoundly affect subjective well-being (18). Ultimately, the well-being of college students is not just a measure of personal happiness but a broader reflection of educational policies and institutional support.

### 1.2 Impact of the COVID-19 pandemic on college students’ mental health and well-being

The outbreak of COVID-19 has exerted extensive influence on numerous areas of life. Among these, the mental health and overall well-being of college students have been significantly impacted (19). The unique difficulties arising from the pandemic have led to increased levels of stress, anxiety, and social isolation within this student population (20, 21).

Adapting to online learning was challenging for many students due to the lack of personal interaction compared to traditional classrooms (22). They faced issues like unreliable internet and navigating various online platforms for classes, assignments, and exams, which created immense pressure (23). The absence of face-to-face contact with peers and professors also led to increased feelings of loneliness and isolation (24). Additionally, the pandemic caused heightened anxiety among students due to uncertainties in their lives (25). The academic environment became unpredictable, with institutions changing grading policies, exam methods, or class formats at short notice, further increasing anxiety (26). Moreover, the cancelation or remote conversion of internships and fewer job opportunities added to the academic and career-related uncertainties (27).

During the pandemic, social isolation became a significant issue (28). This separation disrupted the crucial social connections that are vital to college life and personal development (83). For students predisposed to mental health issues, the lack of social support significantly jeopardized their overall well-being (29). Additionally, they encountered numerous domestic stressors, such as suboptimal study conditions, increased household duties, and familial discord (30).

Some people adapted by creating new routines, doing physical exercise, or practicing mindfulness and meditation (31, 32). However, others had difficulty maintaining a balanced lifestyle. The heavy use of digital communication also caused potential information overload (33). Constant updates on the pandemic increased feelings of helplessness and fear (34), and social media has affected students’ well-being (35). Despite these challenges, universities have improved mental health support through virtual counseling, workshops, and peer programs, which will likely influence future practices in supporting student well-being.

### 1.3 Rationale and objectives of the systematic review

The COVID-19 pandemic has significantly reshaped the experiences of Chinese college students (36–38). This global health crisis has exerted a substantial influence on various dimensions of their lives, such as educational practices, social engagement, financial conditions, and psychological well-being. Subjective well-being, which reflects an individual’s self-assessment of happiness and life satisfaction, is a vital indicator. It is closely associated with mental health, academic performance, and long-term life satisfaction.

Before the pandemic, the subjective well-being of Chinese college students was shaped by a complex interplay of factors, including academic stress, family expectations, personal relationships, and societal pressures (84). The highly competitive academic environment in China, coupled with the significant pressure to achieve academic success, often led to stress and anxiety among students (39). These stressors were further exacerbated by family expectations and societal norms, which placed additional demands on students to conform to culturally prescribed roles and responsibilities (84). Moreover, personal relationships, encompassing friendships and romantic relationships, played a crucial role in influencing students’ overall experiences and subjective well-being (40). The outbreak of the COVID-19 pandemic introduced substantial disruptions to these aspects of students’ lives. The transition of educational institutions to online learning platforms, while necessary for social distancing, presented new challenges for students (22). The absence of in-person interactions hindered some students’ ability to remain engaged in their studies (23). Additionally, the blurring of boundaries between home and educational environments contributed to increased stress levels (41). The economic fallout from the pandemic also cast uncertainty over students’ futures, with job prospects and financial security emerging as major sources of concern (27).

After the pandemic, a notable enhancement in the resilience and adaptability of students has been observed (42). In this context, it is imperative to conduct a comprehensive investigation into the various factors that influence subjective well-being. This review aims to generate meaningful insights and provide robust support, thereby contributing to the improvement of the overall well-being among Chinese college students in the post-pandemic period.

### 1.4 Research questions

To address these literature gaps, this systematic review synthesizes current empirical evidence to answer two primary research questions:

(RQ1) What are the core predictors associated with subjective well-being among Chinese college students?

(RQ2) How do these identified predictors differ across the pre-pandemic, peri-pandemic, and post-pandemic periods?

By answering these questions, this study aims to provide a reliable evidence base for developing targeted student mental health interventions.

## 2 Methods

This study conducted the final database search on December 30, 2024. To ensure data quality, we applied specific search filters to include only English-language articles published in peer-reviewed journals. Two researchers independently managed the entire study selection process. They first screened the titles and abstracts, and then evaluated the full texts of eligible papers. Following the selection phase, these same two reviewers independently performed data extraction and thematic coding. We utilized an inductive approach to derive core themes directly from the literature rather than relying on a pre-existing framework. Whenever discrepancies emerged during the screening or coding stages, the reviewers discussed the issues to reach a mutual consensus. The analytical process followed a two stage thematic synthesis to maintain methodological precision. During the initial extraction phase, a deductive approach was used to identify findings that specifically addressed the research objectives. These extracted findings were then synthesized through an inductive process that allowed core themes to emerge organically from across the literature. This dual strategy acknowledges that the original studies already involved some degree of interpretation by their authors. By integrating these two stages, the researchers successfully organized the evidence into a new framework that remains firmly grounded in the synthesized data.

### 2.1 Search strategy and criteria

In this review, the systematic review methodology from Page et al. (43) was employed to locate, evaluate, and integrate empirical studies concerning subjective well-being. A thorough search strategy was implemented across PubMed, Web of Science, and Scopus databases. For inclusion, studies had to be published in English, within a specified timeframe, in peer-reviewed journals, and closely related to the research topic. Studies that were not relevant to the topic were excluded, thereby maintaining the precision and focus of the analysis.

A literature search was conducted to identify empirical studies on the subjective well-being of Chinese college students in the periods before and after the COVID-19 outbreak. Following the PRISMA 2020 framework to maximize methodological transparency and reproducibility, a comprehensive search was executed across PubMed, Web of Science, and Scopus, with the final retrieval completed on December 30, 2024. The search strategy employed the Boolean operator “AND” to intersect core thematic clusters and population identifiers; specifically, the exact search string used was (“subjective well-being”) AND (“Chinese college students”). Inclusion was strictly governed by predefined filters: empirical studies published in peer-reviewed English-language journals that utilized subjective well-being as the primary research variable among Chinese college students. Conversely, this review excluded non-empirical research, non-English publications, and gray literature—including manuscripts, reports, and conference papers—as well as studies involving non-Chinese student populations or those where subjective well-being served only as a secondary variable. By explicitly documenting these Boolean logical operators, the precise search strings, all applied filters, and the definitive search date, this revised section ensures the review is fully reproducible and methodologically transparent.

Articles published between 2018 and 2024 were reviewed. To ensure comprehensive coverage of the extant literature, a thorough search was conducted using keywords related to subjective well-being (e.g., life satisfaction, happiness, positive affect). This method helped compile a collection of relevant studies for analysis. A total of 518 articles were identified. To ensure transparency and mitigate bias, the search and selection process was documented. Duplicates were removed and the most recent, comprehensive reports were selected. The inclusion and exclusion criteria are detailed in Table 1.

**Table 1:** Inclusion and exclusion criteria.

No.	Inclusion criteria	Exclusion criteria
1	Empirical studies	Non-empirical studies
2	Published in English language	Published in non-English language
3	Peer-reviewed journal	Manuscripts, reports and conference papers
4	Chinese college students	Non-Chinese college students
5	Subjective well-being as the primary research variable	Subjective well-being as a secondary variable

### 2.2 Study selection procedures

**Figure 1:**
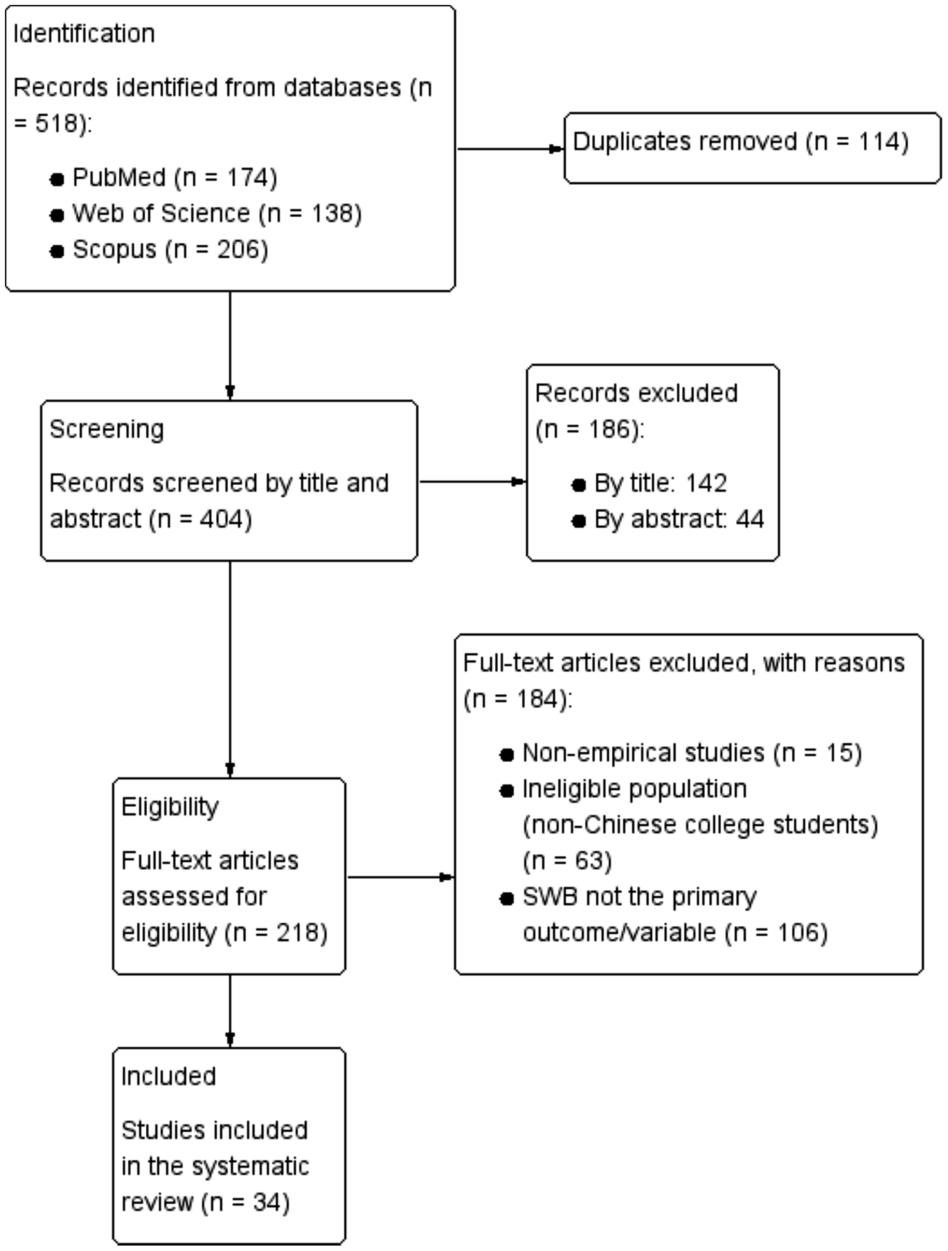
PRISMA flow diagram made by software Review Manager 5.3.

This study followed the PRISMA guidelines, which include 27 items covering various aspects of systematic reviews. A total of 34 eligible articles were selected, with 19 from PubMed, 8 from Web of Science, and 7 from Scopus, as shown in Figure 1.

### 2.3 Data extraction and quality assessment

Data extraction is crucial in systematic reviews. The first author extracted the data into a shared Excel spreadsheet, which was then verified by the second author. The extraction table included publication characteristics, author details, study design, and baseline research methodology. The selection process for the study is illustrated in Figure 1, which presents a PRISMA flow diagram.

The study design was assessed using the Joanna Briggs Institute (JBI) Critical Appraisal Checklists, specifically the checklist for cohort studies (44), quasi-experimental studies (45), qualitative studies (46), and analytical cross-sectional studies. Following the protocol of the JBI Collaboration, two independent reviewers performed a rigorous methodological appraisal using the appropriate JBI Critical Appraisal Checklists tailored to each study design. To maintain methodological consistency, each included paper was subjected to a dual-reviewer appraisal process using JBI-standardized tools. While the number of appraisal criteria was specific to the research design (e.g., 8 items for cross-sectional and 10 items for qualitative research), the assessment consistently focused on key quality markers including inclusion criteria, contextual detail, and the reliability of measurement instruments. Any inter-reviewer discrepancies were resolved through deliberative consensus to ensure the integrity of the quality grading. Quality assessment followed a normalized scoring system to account for variations in appraisal instruments (8 to 11 total scores). A tiering strategy was implemented where a compliance rate of ≥80% signified high methodological quality. Moderate and low-quality designations were reserved for studies meeting 50–79% and less than 50% of the appraisal items, ensuring cross-tool comparability.

The methodological integrity of the 34 included studies was rigorously evaluated through the implementation of a diversified appraisal framework tailored to the specific nuances of each research design. By utilizing four distinct JBI checklists, this approach captured critical design-specific elements that a singular instrument might have overlooked, including congruity in qualitative methodologies, confounding control in cross-sectional analyses, and retention reliability in cohort studies. This multi-instrument strategy facilitated a standardized normalization of quality through percentage-based thresholds, which enabled a coherent comparison of evidentiary strength across varying levels of methodological complexity. Ultimately, the results establish a robust foundation for the subsequent synthesis, as the majority of the included literature demonstrated high compliance with their respective appraisal criteria and thus reinforced the overall credibility of the findings (Table 2).

**Table 2:** Methodological quality assessment summary (*N* = 34).

ID	Author (year)	Study design	Appraisal tool applied	Total score	Quality
1	Cheng et al. (49)	Cross-Sectional	JBI Analytical Cross-Sectional	8/8	High
2	Li et al. (71)	Cross-Sectional	JBI Analytical Cross-Sectional	8/8	High
3	Yang and Wang (50)	Cross-Sectional	JBI Analytical Cross-Sectional	8/8	High
4	Xue et al. (73)	Diary Study	JBI Cohort Studies	11/11	High
5	Yuan and You (61)	Cross-Sectional	JBI Analytical Cross-Sectional	8/8	High
6	Qiu et al. (72)	Cross-Sectional	JBI Analytical Cross-Sectional	8/8	High
7	Cheng et al. (40)	Longitudinal	JBI Cohort Studies	7/11	Moderate
8	Zhang et al. (62)	Cross-Sectional	JBI Analytical Cross-Sectional	8/8	High
9	Dong and Ni (65)	Cross-Sectional	JBI Analytical Cross-Sectional	6/8	Moderate
10	Ju et al. (66)	Cross-Sectional	JBI Analytical Cross-Sectional	8/8	High
11	Su and He (67)	Cross-Sectional	JBI Analytical Cross-Sectional	8/8	High
12	Wu et al. (68)	Cross-Sectional	JBI Analytical Cross-Sectional	8/8	High
13	Feng and Yang (69)	Cross-Sectional	JBI Analytical Cross-Sectional	6/8	Moderate
14	Zuo et al. (60)	Cross-Sectional	JBI Analytical Cross-Sectional	8/8	High
15	Xu et al. (57)	Qualitative	JBI Qualitative Research	8/10	High
16	Jiang et al. (74)	Cross-Sectional	JBI Analytical Cross-Sectional	6/8	Moderate
17	Wang et al. (75)	Cross-Sectional	JBI Analytical Cross-Sectional	8/8	High
18	Chen et al. (77)	Cross-Sectional	JBI Analytical Cross-Sectional	8/8	High
19	Wang et al. (52)	Longitudinal	JBI Cohort Studies	11/11	High
20	Huang and Zhang (55)	Cross-Sectional	JBI Analytical Cross-Sectional	8/8	High
21	Zhang et al. (34)	Cross-Sectional	JBI Analytical Cross-Sectional	8/8	High
22	Wang (64)	Cross-Sectional	JBI Analytical Cross-Sectional	6/8	Moderate
23	Hou et al. (53)	Mixed (CS & Qual)	JBI Qualitative Research	7/10	Moderate
24	Ran et al. (51)	Longitudinal	JBI Cohort Studies	11/11	High
25	Xu et al. (78)	Cross-Sectional	JBI Analytical Cross-Sectional	6/8	Moderate
26	Yu et al. (80)	Cross-Sectional	JBI Analytical Cross-Sectional	8/8	High
27	Zhang et al. (81)	Cross-Sectional	JBI Analytical Cross-Sectional	8/8	High
28	Wang and Fu (56)	Cross-Sectional	JBI Analytical Cross-Sectional	6/8	Moderate
29	Lan and Wang (76)	Cross-Sectional	JBI Analytical Cross-Sectional	8/8	High
30	Liu et al. (79)	Quasi-Exp	JBI Quasi-Experimental	9/9	High
31	Liu et al. (54)	Cross-Sectional	JBI Analytical Cross-Sectional	8/8	High
32	Zhao (59)	Cross-Sectional	JBI Analytical Cross-Sectional	8/8	High
33	Ye et al. (58)	Cross-Sectional	JBI Analytical Cross-Sectional	8/8	High
34	Bi and Li (70)	Cross-Sectional	JBI Analytical Cross-Sectional	6/8	Moderate

Methodological quality was evaluated using four study-specific JBI critical appraisal checklists: 8 items for analytical cross-sectional studies, 9 items for quasi-experimental research, 10 items for qualitative studies, and 11 items for cohort/longitudinal designs. To ensure cross-tool comparability, quality ratings were standardized via percentage thresholds: High Quality (≥ 80%), Moderate Quality (50–79%), and Low Quality (< 50%).

### 2.4 Ethics and consent statements

The ethical approval and consent to participate that would typically form the basis of research studies are not applicable in this particular study. It is imperative to note that all authors have provided their informed consent for the publication of this study.

## 3 Results

### 3.1 Overview of included studies

The search and review results, including the number of studies, are presented here to show the review’s scope. More studies indicate a comprehensive exploration, while fewer suggest a focused examination (47, 48). The details are in Table 3. Subjective well-being, a complex construct encompassing life satisfaction and emotions (2), is measured using various scales and questionnaires. Differences in these measures affect research conclusions and comparability.

**Table 3:** Summary of included studies.

ID	Author (year)	Aim of study	Design	Participants (N)	Primary variables
1	Cheng et al. (49)	Love forgiveness and SWB	Cross-sectional	831	Love Forgiveness, Interpersonal Relationships
2	Li et al. (71)	Family culture, personality, and SWB	Cross-sectional	340	Neuroticism, Extraversion, Family culture
3	Yang and Wang (50)	Family rituals and mental health	Cross-sectional	424	Family rituals, Cohesion, Adaptability
4	Xue et al. (73)	Leisure crafting and well-being	Diary study	80	Leisure resources, Social leisure
5	Yuan and You (61)	Physical activity during pandemic	Cross-sectional	1,198	Physical Activity, Adverse mental health
6	Qiu et al. (72)	Labor education and SWB	Cross-sectional	2,028	Labor education, Self-efficacy, Lifestyle
7	Cheng et al. (40)	Pandemic policies and behavior	Longitudinal	1,641	Love forgiveness, Interpersonal relationships
8	Zhang et al. (62)	Physical activity and perceived health	Cross-sectional	1,204	MVPA guideline, Perceived health
9	Dong and Ni (65)	Dispositional awe and SWB	Cross-sectional	332	Awe, Openness, Extraversion
10	Ju et al. (66)	Strength-based parenting and growth	Cross-sectional	621	Parenting, Personal growth, Strengths use
11	Su and He (67)	Sleep quality and resilience	Cross-sectional	3,349	Sleep quality, Resilience, Just world belief
12	Wu et al. (68)	Child maltreatment and self-esteem	Cross-sectional	358	Maltreatment, Self-esteem, Self-compassion
13	Feng and Yang (69)	Achievement motivation and control	Cross-sectional	1,017	Motivation, Self-control, Self-management
14	Zuo et al. (60)	Character strengths and social support	Cross-sectional	336	Character strengths, Support, Emotions
15	Xu et al. (57)	Social media and experiential happiness	Qualitative	5	Social media use, Identity, Comparison
16	Jiang et al. (74)	Coping styles and emotion regulation	Cross-sectional	1,127	Coping styles, Emotion regulation
17	Wang et al. (75)	Ambivalence over emotional expression	Cross-sectional	555	Fear of intimacy, Attachment avoidance
18	Chen et al. (77)	Exercise adherence and character	Cross-sectional	1,001	Exercise adherence, Mental character
19	Wang et al. (52)	SNS usage and psychological well-being	Longitudinal	265	Passive Social Networking Site Usage
20	Huang and Zhang (55)	Social support in online learning	Cross-sectional	515	Social support, Psychological capital
21	Zhang et al. (34)	Physical activity intensity	Cross-sectional	723	Vigorous/Moderate physical activity
22	Wang (64)	Trait gratitude and basic needs	Cross-sectional	481	Trait gratitude, Psychological needs
23	Hou et al. (53)	Religiosity and meaningfulness	Mixed (CS & Qual)	1,418	Religiosity, Meaningfulness
24	Ran et al. (51)	Career adjustment and calling	Longitudinal	1,077	Career Exploration/Calling, Self-reflection
25	Xu et al. (78)	Career capital effect on well-being	Cross-sectional	312	Career capital, Career adaptability
26	Yu et al. (80)	Longitudinal trajectories of SWB	Longitudinal	1,050	Self-determination, Resilience factors
27	Zhang et al. (81)	Sport anxiety and need satisfaction	Cross-sectional	835	Sport anxiety, Need satisfaction
28	Wang and Fu (56)	Internet addiction and social support	Cross-sectional	681	Internet addiction, Meaning in life
29	Lan and Wang (76)	Socio-economic status and values	Cross-sectional	600	Values, Socio-economic status
30	Liu et al. (79)	Education expansion and SWB	Quasi-exp	15,301	Higher Education Expansion policy
31	Liu et al. (54)	Authoritarian personality	Cross-sectional	1,007	Authoritarianism, Organizational culture
32	Zhao (59)	Social media addiction	Cross-sectional	3,370	Social media use/addiction
33	Ye et al. (58)	Social anxiety and emotional efficacy	Cross-sectional	908	Social anxiety, Emotional self-efficacy
34	Bi and Li (70)	Psychological flexibility profiles	Cross-sectional	644	Psychological flexibility, Adjustment

The 34 empirical studies incorporated within the review encompassed a variety of research designs, with 25 studies (73.5%) categorized as ‘high quality’ and 9 studies (26.4%) as ‘moderate quality’. Despite the preponderance of cross-sectional designs in the corpus, a significant proportion of high-scoring papers (e.g., (49, 50)) have demonstrated exceptional rigor in their utilization of validated instruments such as the Satisfaction with Life Scale (SWLS) and the incorporation of salient demographic variables. The high degree of methodological consistency across the 34 studies provides a reliable ‘snapshot’ of the factors influencing subjective well-being during and after the pandemic.

The longitudinal and quasi-experimental evidence offers strong support for the causal pathways influencing student well-being. Weekly assessments demonstrate that active engagement in leisure crafting leads to significant fluctuations in happiness over time. Research utilizing multi-wave designs further identifies a reciprocal link between passive social media use and psychological outcomes, indicating that digital habits and mental health are mutually reinforcing. Moreover, longitudinal data show that career exploration and a sense of calling sequentially enhance vocational adaptability and overall life satisfaction. Large-scale policy evaluations using quasi-experimental methods also confirm that structural shifts, such as educational expansion, significantly impact happiness during major social transitions. Complementing these quantitative trends, qualitative and comparative analyses provide essential context regarding the subjective nature of student experiences. Interpretative investigations reveal a persistent struggle between social media indulgence and the desire for digital temperance among university students. Evidence from mixed-methods research suggests that religious beliefs bolster well-being specifically by providing a framework for personal meaningfulness. Furthermore, comparative studies highlight how macro-level events, such as the COVID-19 pandemic, have reshaped interpersonal dynamics and emotional forgiveness over several years. These non-cross-sectional findings collectively illustrate the intricate interpretations and long-term adjustments students make in response to both personal and environmental changes.

Evidently, psychological flexibility and internal resilience emerged as the primary defensive anchors; high-quality evidence underscores that cognitive resources—specifically trait gratitude and self-compassion—served as vital psychological buffers during the pandemic’s most volatile phases. Moving beyond the individual level, the data highlights socio-familial dynamics as a second indispensable cluster. Crucially, the integration of longitudinal observations (e.g., (51, 52)) clarifies that family rituals and interpersonal cohesion are not just correlates but enduring stabilizers that sustained happiness throughout the transition to the post-pandemic era.

The synthesis indicates that behavioral adjustments, particularly physical activity and sleep hygiene, are consistently associated with emotional stability across both qualitative insights and large-scale empirical datasets (*N* = 15,301). While systemic elements, such as shifting educational policies, certainly delineate the student landscape, our JBI-led appraisal of high-quality evidence underscores that personal adaptive capacity remains the decisive internal conduit for subjective well-being. These results suggest that studies which employ robust methodologies place significant emphasis on individual adaptive capacity as a key predictor of subjective well-being. In the context of these findings, the concept of well-being can be understood as a dynamic outcome resulting from the continuous interaction between environmental pressures and internal coping mechanisms.

### 3.2 Differences in subjective well-being before and after the COVID-19 pandemic

In this pre-pandemic period, subjective well-being among college students was largely influenced by traditional stressors, such as academic pressure and career planning, often mitigated by the supportive presence of peer networks and faculty guidance (52–54). Following the onset of the pandemic, significant shifts occurred in their subjective well-being. For Chinese college students, the sudden shift to remote learning brought new challenges such as technological anxiety and reduced face-to-face interactions (55–60).

Despite these challenges, some adaptive mechanisms emerged among students. Some promote improvements in subjective well-being through love (49). Some improved subjective well-being levels through sport, meaning that participation in moderate to high levels of physical activity was effective in mitigating the negative psychological impact of the COVID-19 pandemic (61–63).

### 3.3 Factors influencing the differences in subjective well-being

Based on a systematic review of the literature, this study finds that the subjective well-being of Chinese college students is influenced by personality traits, mental health, family and educational background, social support, interpersonal relationships, physical activity, healthy lifestyle, psychological state, coping strategies, and socio-cultural and economic factors.

Firstly, personality traits play a significant role in influencing well-being. For example, Wang (64) emphasized the importance of gratitude in promoting happiness. Research by Li et al. (19) indicated that neuroticism tends to diminish happiness levels, whereas extraversion generally enhances them. Dong and Ni (65) demonstrated that emotional regulation plays a crucial role in mediating this relationship. Additionally, Ju et al. (66) and Su and He (67) delved into how resilience and belief in a just world can affect happiness. Wu et al. (68) and Feng and Yang (69) established connections between self-esteem and achievement motivation and overall well-being. Bi and Li (70) and Cheng et al. (49) examined the contributions of psychological flexibility and forgiveness to happiness.

Secondly, both family and educational factors have profound impacts on well-being (71). Yang and Wang (50) revealed that family rituals are closely associated with the mental health of adolescents. Qiu et al. (72) discovered that labor education can enhance self-efficacy and promote healthy lifestyles among college students. Cheng et al. (40) and Ran et al. (51) explored the connections between family dynamics and career exploration, and their relevance to well-being. Xu et al. (57) also highlighted the influence of social media on happiness, which has important implications for educational contexts.

Subsequently, social support and relationships matter for well-being. Xue et al. (73) highlighted that engaging in leisure activities can effectively boost happiness. Meanwhile, a series of studies have underscored the positive impact of strong social networks and positive relationships on happiness, including those by Jiang et al. (74), Wang et al. (75), Lan and Wang (76), Zhao (59), and Huang and Zhang (55). In addition to social factors, physical exercise and healthy living have also been proven to enhance well-being (77). Yuan and You (61) and Zhang et al. (34) demonstrated that regular physical activity is beneficial for well-being, while Cheng et al. (49) and Zhang et al. (62) emphasized the importance of maintaining a healthy diet and sufficient sleep for achieving greater happiness.

Moreover, the way we handle stress has a significant impact on our happiness. Wang (64) and Hou et al. (53) investigated how different coping styles shape well-being. Ye et al. (58) explored how emotional self-efficacy and a sense of life meaning can influence happiness. Jiang et al. (74) further highlighted the role of emotion regulation in effective coping strategies.

Ultimately, the influence of sociocultural and economic factors on happiness is extensive and multifaceted. For example, Xu et al. (78) have demonstrated a clear link between career capital and overall well-being, highlighting how economic opportunities and professional development can shape one’s sense of happiness. Liu et al. (79) have delved into the long-term effects of higher education policies, showing how these policies can have a lasting impact on individuals’ happiness by influencing their educational and career trajectories. Yu et al. (80) have explored the role of self-determination in goal pursuit, revealing that the cultural emphasis on autonomy and personal goals can significantly affect happiness. Wang and Fu (56) and Zhang et al. (81) have also examined how modern lifestyle factors such as internet use and sports anxiety can influence happiness, reflecting the broader sociocultural and economic contexts that shape our daily experiences and well-being.

## 4 Discussion

The credibility of this systematic review is fundamentally rooted in the methodological integrity of the constituent studies. Rather than relying on reductive numerical scores, study quality was evaluated through the JBI Critical Appraisal Checklist, prioritizing a nuanced assessment of specific reporting domains. While some literature exhibited gaps in accounting for confounding variables, the majority of the included studies demonstrated high procedural consistency. Specifically, these primary sources adhered to stringent protocols regarding participant recruitment, utilized psychometrically validated subjective well-being (SWB) scales, and implemented robust statistical frameworks. Although the prevalence of cross-sectional designs remains a structural limitation within the field, the consistent rigor observed in these core methodological areas reinforces the overall reliability of the synthesized evidence.

A salient critique within post-pandemic psychological discourse concerns the over-reliance on cross-sectional data, an approach that inherently restricts the capacity to draw definitive causal inferences between specific COVID-19 milestones and subsequent shifts in student well-being. However, our systematic evaluation demonstrates that the most methodologically sound studies [e.g., (49, 50)] moved beyond mere descriptive reporting by identifying and statistically adjusting for critical “nuisance” variables, such as socio-economic background, gender, and pre-existing mental health status. By addressing these potential biases through multivariate techniques, the synthesized evidence provides a more nuanced and reliable “snapshot” of the determinants of university students’ happiness than is typically afforded by simpler descriptive surveys.

Furthermore, the strategic integration of longitudinal datasets with high quality scores (e.g., (51, 52)) provides this synthesis with a necessary dimension of temporal depth. The alignment between these longitudinal observations and the broader cross-sectional pool—particularly concerning the enduring influence of social support, familial cohesion, and individual resilience—suggests that the identified factors are persistent predictors of well-being rather than transient artifacts of the pandemic environment. Consequently, the methodological discipline observed across these 34 studies permits a confident and robust interpretation of the psychological landscape for university students in the post-pandemic era.

### 4.1 Implications for theory

According to self-determination theory (82), the three basic psychological needs of autonomy, competence and relatedness underlie individual behavior and have a profound influence on well-being.

From the perspective of self-determination theory (82), six factors influence Chinese college students’ subjective well-being and work together. Personality traits and mental health affect students’ autonomy. Good mental health allows students to make decisions aligned with their values and interests. Positive personality traits help build healthy relationships, fulfilling the need for relatedness. Family and educational background meet autonomy and competence needs through emotional support and educational resources. Democratic parenting and high-quality education further enhance these aspects. Social support and interpersonal relationships provide emotional comfort and practical help, meeting relatedness needs and promoting competence through cooperation and communication. Physical activity and a healthy lifestyle reflect autonomous health management. They fulfill autonomy needs while improving physical fitness and psychological resilience, which also meets competence needs. Psychological state and coping strategies directly impact reactions to life events and emotional experiences. Positivity and effective coping help handle stress and meet all three basic needs. Socio-cultural and economic factors provide the external environment and material basis. A positive socio-cultural atmosphere satisfies relatedness needs, while a stable economic environment with moderate financial support reduces anxiety and indirectly influences subjective well-being.

This study findings are in alignment with the principles of self-determination theory (82). This correspondence provides substantial empirical evidence in support of the application of the theory among college students.

### 4.2 Implications for policy and practice

In the aftermath of the pandemic, its lasting impact on college students highlights the necessity for policy and practice adjustments. Policymakers should allocate resources to enhance mental health services and maintain advanced online platforms. Reducing stigma and ensuring accurate public health information remain essential. Educational institutions must promote mental health awareness, train faculty to detect early signs of distress, and offer flexible learning options like hybrid or fully online courses. These measures can create a more resilient and adaptable educational environment.

Mental health professionals play a crucial role in the post-pandemic context. They need to develop targeted interventions for students’ unique challenges. Virtual sessions and peer support networks should continue to be utilized. Training in telehealth and ensuring access to services regardless of location are vital. Integrating mental health screenings into university health services can aid in early intervention. Collaboration among policymakers, educational institutions, and mental health professionals is key to building a comprehensive support system for college students.

### 4.3 Limitations of the review

Several methodological constraints within this systematic review warrant careful consideration. First, the exclusion of non-English publications and gray literature, including conference papers and technical reports, presents a potential for both linguistic and publication biases within the findings. Second, although design-specific JBI critical appraisal instruments were applied to maintain evaluative rigor across various methodologies, the evidence base is primarily composed of cross-sectional research. This structural characteristic limits the ability to establish definitive causal pathways because such studies provide only a temporal snapshot of the phenomena under investigation. While the inclusion of sophisticated longitudinal and quasi-experimental designs, specifically Studies 4, 7, 15, 19, 23, 24, and 30, offers more robust insights into behavioral and psychological evolution, the overall certainty of the synthesized evidence remains influenced by the inherent limitations of the primary data. Finally, the absence of prospective registration in an international protocol database, such as PROSPERO, is recognized as a procedural limitation that impacts the full transparency of the research process.

### 4.4 Recommendations for future research

The comprehensive review of existing literature has illuminated the current research landscape, while also revealing several gaps and limitations that offer promising avenues for future exploration. Future longitudinal studies should examine the causal pathways linking family cohesion identified here as a key predictor to SWB outcomes in the post-pandemic campus environment. Secondly, subsequent interventional research should evaluate how university mental health initiatives can effectively integrate this family-based support to sustain student resilience over time. A crucial area for further investigation is the exploration of mechanisms through detailed empirical studies and the refinement of methodologies. Future work might employ experimental or longitudinal studies to gain a deeper understanding of causality. Increasing sample diversity and utilizing rigorous sampling techniques can enhance the robustness of research. Advanced data analysis and mixed-methods approaches can produce more accurate and reliable results. Interdisciplinary collaborations, as well as partnerships with industry and policymakers, can drive innovation and ensure that research findings are applicable in real-world contexts.

Moreover, the review has also emphasized the significance of long-term monitoring and evaluation. Many studies currently lack follow-up assessments that could track the sustainability of observed effects over extended periods. Designing studies with multiple follow-up phases can provide valuable insights into the persistence of outcomes and the factors that influence long-term success. These insights are essential for developing interventions and policies aimed at creating lasting positive impacts. Additionally, increasing engagement with community stakeholders and end-users of research should be a priority. Participatory approaches that involve stakeholders as active participants in the research process can ensure that the research addresses real-world needs and priorities. This engagement can also facilitate the dissemination and application of research findings, as stakeholders are more likely to adopt strategies and interventions when they have been involved in their development.

Lastly, the ethical considerations of research must not be neglected. Ensuring that studies adhere to the highest ethical standards is crucial for maintaining public trust and the integrity of scientific inquiry. This includes obtaining informed consent, safeguarding participant privacy, and being transparent about potential conflicts of interest. Researchers should also aim to communicate their findings in a manner that is accessible to a broad audience, thereby promoting greater public understanding and support for scientific endeavors. To advance knowledge, future research must be comprehensive, innovative, and ethical. Addressing the current limitations will generate valuable insights and practical solutions, ultimately benefiting both academia and society.

## 5 Conclusion

The synthesized evidence directly answers the core inquiries. For the first research question (RQ1), the data indicate that social support, physical activity, and internal resilience serve as the most consistent predictors of student well-being. However, the influence of these variables was not static (RQ2). Before the outbreak, typical academic and social dynamics largely determined psychological outcomes. This baseline shifted drastically during the pandemic’s peak, as isolation, screen fatigue, and health anxieties became major risk factors. Moving into the recovery phase, the literature reveals yet another transition: family cohesion and active coping strategies proved to be the critical elements for long-term emotional stability.

In conclusion, the factors influencing the differences in Chinese college students’ subjective well-being before and during the COVID-19 pandemic are multifaceted, including personality traits and mental health, family and educational background, social support and interpersonal relationships, physical activity and healthy lifestyle, as well as psychological state and coping strategies. Addressing these factors is crucial for students’ holistic development and success. Educational institutions can play a pivotal role by implementing comprehensive strategies and fostering supportive environments to safeguard students’ mental health and well-being. The lessons learned during the pandemic can inform the development of more resilient and adaptive educational frameworks, ultimately contributing to a healthier and more supportive learning environment for future generations.

## Data Availability

The original contributions presented in the study are included in the article/Supplementary material, further inquiries can be directed to the corresponding author.
