# Supplementary material for: A systematic review of predictors of college students’ subjective well-being: evidence from pre- and post-pandemic literature
Source: Front Public Health. 2026 Apr 16;14:1793063. doi: 10.3389/fpubh.2026.1793063 (PMC13128366; doi:10.3389/fpubh.2026.1793063)
Supplement: Supplementary file 1 [file Table_1.docx]

| **Section and Topic** | **Item #** | **Checklist item** | **Location where item is reported** |
| --- | --- | --- | --- |
| **TITLE** | | |  |
| Title | 1 | A Systematic Review of Predictors of College Students' Subjective Well-being: Evidence from Pre- and Post-pandemic Literature | 1 |
| **ABSTRACT** | | |  |
| Abstract | 2 | The checklist includes the following 12 items   1. Identify the report as a systematic review 2. Provide an explicit statement of the main objective(s) or question(s) the review addresses 3. Specify the inclusion and exclusion criteria for the review 4. Specify the information sources (such as databases, registers) used to identify studies and the date when each was last searched 5. Specify the methods used to assess risk of bias in the included studies 6. Specify the methods used to present and synthesise results 7. Give the total number of included studies and participants and summarise relevant characteristics of studies 8. Present results for main outcomes, preferably indicating the number of included studies and participants for each. If meta-analysis was done, report the summary estimate and confidence/credible interval. If comparing groups, indicate the direction of the effect (that is, which group is favoured) 9. Provide a brief summary of the limitations of the evidence included in the review (such as study risk of bias, inconsistency, and imprecision) 10. Provide a general interpretation of the results and important implications 11. Specify the primary source of funding for the review 12. Provide the register name and registration number | 1 |
| **INTRODUCTION** | | |  |
| Rationale | 3 | According to self-determination theory (Ryan & Deci, 2017), the three basic psychological needs of autonomy, competence and relatedness underlie individual behaviour and have a profound influence on well-being. | 3 |
| Objectives | 4 | To investigate the factors affecting changes in subjective well-being among Chinese college students before and after the pandemic outbreak | 3 |
| **METHODS** | | |  |
| Eligibility criteria | 5 | For the review, inclusion criteria were set to include empirical studies published in English language, peer-reviewed journals, focusing on Chinese college students, and where subjective well-being was the primary research variable. On the other hand, exclusion criteria excluded non-empirical studies, those published in non-English languages, manuscripts, reports, and conference papers, studies involving non-Chinese college students, and those where subjective well-being was not the primary research variable. Studies were grouped for syntheses based on these criteria to ensure a focused and relevant analysis of the data pertaining to Chinese college students' subjective well-being. | 3 |
| Information sources | 6 | A retrospective review of articles published between 2018 and 2024 was conducted, employing a comprehensive search strategy across the PubMed, Web of Science, and Scopus databases. | 4 |
| Search strategy | 7 | The search term employed was "subjective well-being". | 4 |
| Selection process | 8 | A total of 518 relevant studies were successfully compiled for analysis, including 174 retrieved from the PubMed database, 138 from the Web of Science database, and 206 from the Scopus database. | 4 |
| Data collection process | 9 | Two authors processed the 518 articles collected. They applied the inclusion and exclusion criteria outlined in point five. The information from the screened articles was listed in an Excel spreadsheet. | 4 |
| Data items | 10a | The articles included in this collection were published between 2018 and 2024. The subsequent step involves the removal of duplicate articles. Subsequently, a search should be conducted using the criteria of "title and abstract" in order to eliminate articles that are not related to the subject of subjective well-being in college students. In the subsequent phase of the research, non-empirical studies and articles in which the research subjects were not Chinese college students will be eliminated. In conclusion, it is imperative to retain articles that treat subjective well-being as the primary research variable. | 5 |
|  | 10b | For inclusion, studies had to be published in English, within a specified timeframe, in peer-reviewed journals, and closely related to the research topic. | 5 |
| Study risk of bias assessment | 11 | After retrieving 518 articles from three databases, they were exported to Excel. Two authors independently re-screened suitable articles based on the fifth criterion using the Excel file. The work progress of both authors was presented through online updates in Excel, thereby eliminating redundant work and ensuring independent task execution. | 5 |
| Effect measures | 12 | Empirical research was employed, with P < 0.05 indicating statistical significance. | 5 |
| Synthesis methods | 13a | Summarize the title, abstract, and study subjects to confirm compliance with the inclusion criteria outlined in item five. | 5 |
|  | 13b | Check whether the screened articles have missing data, and whether the checks for missing data meet the inclusion criteria. | 5 |
|  | 13c | Inductive Analysis | 5 |
|  | 13d | Review Manager 5.3 | 5 |
|  | 13e | Descriptive statistics, inductive analysis. | 5 |
|  | 13f | A comparison of fixed-effects and random-effects models was conducted, or subgroup analyses were performed to examine the effects in specific populations. | 5 |
| Reporting bias assessment | 14 | All included studies underwent risk assessment by two independent evaluators following standardized scoring guidelines. Discrepancies in assessment results were discussed and resolved through consensus. | 5 |
| Certainty assessment | 15 | Two authors independently screened studies based on inclusion criteria, discussed contentious items, and confirmed agreement on consistency, directness, precision, study design, and risk of bias.  **Table 2** Table 2 Methodological Quality Assessment Summary (N = 34)   \| **ID** \| **Author (Year)** \| **Study Design** \| **Appraisal Tool Applied** \| **Total Score** \| **Quality** \| \| --- \| --- \| --- \| --- \| --- \| --- \| \| 1 \| Cheng et al. (2021) \| Cross-Sectional \| JBI Analytical Cross-Sectional \| 8/8 \| High \| \| 2 \| Li et al. (2022) \| Cross-Sectional \| JBI Analytical Cross-Sectional \| 8/8 \| High \| \| 3 \| Yang & Wang (2023) \| Cross-Sectional \| JBI Analytical Cross-Sectional \| 8/8 \| High \| \| 4 \| Xue et al. (2022) \| Diary Study \| JBI Cohort Studies \| 11/11 \| High \| \| 5 \| Yuan & You (2022) \| Cross-Sectional \| JBI Analytical Cross-Sectional \| 8/8 \| High \| \| 6 \| Qiu et al. (2023) \| Cross-Sectional \| JBI Analytical Cross-Sectional \| 8/8 \| High \| \| 7 \| Cheng et al. (2023) \| Longitudinal \| JBI Cohort Studies \| 7/11 \| Moderate \| \| 8 \| Zhang et al. (2021) \| Cross-Sectional \| JBI Analytical Cross-Sectional \| 8/8 \| High \| \| 9 \| Dong & Ni (2020) \| Cross-Sectional \| JBI Analytical Cross-Sectional \| 6/8 \| Moderate \| \| 10 \| Ju et al. (2023) \| Cross-Sectional \| JBI Analytical Cross-Sectional \| 8/8 \| High \| \| 11 \| Su & He (2023) \| Cross-Sectional \| JBI Analytical Cross-Sectional \| 8/8 \| High \| \| 12 \| Wu et al. (2022) \| Cross-Sectional \| JBI Analytical Cross-Sectional \| 8/8 \| High \| \| 13 \| Feng & Yang (2024) \| Cross-Sectional \| JBI Analytical Cross-Sectional \| 6/8 \| Moderate \| \| 14 \| Zuo et al. (2024) \| Cross-Sectional \| JBI Analytical Cross-Sectional \| 8/8 \| High \| \| 15 \| Xu et al. (2024) \| Qualitative \| JBI Qualitative Research \| 8/10 \| High \| \| 16 \| Jiang et al. (2021) \| Cross-Sectional \| JBI Analytical Cross-Sectional \| 6/8 \| Moderate \| \| 17 \| Wang et al. (2022) \| Cross-Sectional \| JBI Analytical Cross-Sectional \| 8/8 \| High \| \| 18 \| Chen et al. (2024) \| Cross-Sectional \| JBI Analytical Cross-Sectional \| 8/8 \| High \| \| 19 \| Wang et al. (2018) \| Longitudinal \| JBI Cohort Studies \| 11/11 \| High \| \| 20 \| Huang & Zhang (2022) \| Cross-Sectional \| JBI Analytical Cross-Sectional \| 8/8 \| High \| \| 21 \| Zhang et al. (2022) \| Cross-Sectional \| JBI Analytical Cross-Sectional \| 8/8 \| High \| \| 22 \| Wang (2020) \| Cross-Sectional \| JBI Analytical Cross-Sectional \| 6/8 \| Moderate \| \| 23 \| Hou et al. (2018) \| Mixed (CS & Qual) \| JBI Qualitative Research \| 7/10 \| Moderate \| \| 24 \| Ran et al. (2023) \| Longitudinal \| JBI Cohort Studies \| 11/11 \| High \| \| 25 \| Xu et al. (2023) \| Cross-Sectional \| JBI Analytical Cross-Sectional \| 6/8 \| Moderate \| \| 26 \| Yu et al. (2024) \| Cross-Sectional \| JBI Analytical Cross-Sectional \| 8/8 \| High \| \| 27 \| Zhang et al. (2024) \| Cross-Sectional \| JBI Analytical Cross-Sectional \| 8/8 \| High \| \| 28 \| Wang & Fu (2024) \| Cross-Sectional \| JBI Analytical Cross-Sectional \| 6/8 \| Moderate \| \| 29 \| Lan & Wang (2024) \| Cross-Sectional \| JBI Analytical Cross-Sectional \| 8/8 \| High \| \| 30 \| Liu et al. (2023) \| Quasi-Exp \| JBI Quasi-Experimental \| 9/9 \| High \| \| 31 \| Liu et al. (2019) \| Cross-Sectional \| JBI Analytical Cross-Sectional \| 8/8 \| High \| \| 32 \| Zhao (2021) \| Cross-Sectional \| JBI Analytical Cross-Sectional \| 8/8 \| High \| \| 33 \| Ye et al. (2021) \| Cross-Sectional \| JBI Analytical Cross-Sectional \| 8/8 \| High \| \| 34 \| Bi & Li (2021) \| Cross-Sectional \| JBI Analytical Cross-Sectional \| 6/8 \| Moderate \| | 8 |
| **RESULTS** | | |  |
| Study selection | 16a | **Figure 1.** PRISMA flow diagram made by software Review Manager 5.3  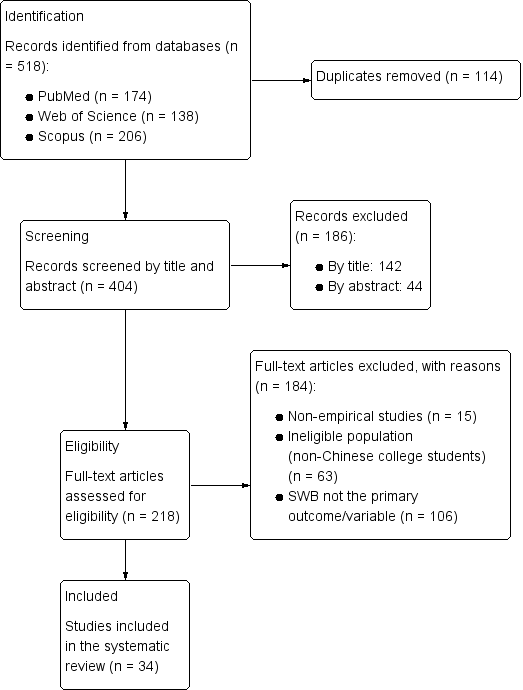 | 6 |
|  | 16b | **Table 1** Inclusion and exclusion criteria   \| **No.** \| **Inclusion criteria** \| **Exclusion criteria** \| \| --- \| --- \| --- \| \| **1** \| Empirical studies \| Non-empirical studies \| \| **2** \| Published in English language \| Published in non-English language \| \| **3** \| Peer-reviewed journal \| Manuscripts, reports and conference papers \| \| **4** \| Chinese college students \| Non-Chinese college students \| \| **5** \| Subjective well-being as the primary research variable \| Subjective well-being as the unprimary research variable \| | 5 |
| Study characteristics | 17 | Based on a systematic review of the literature, this study finds that the subjective well-being of Chinese college students is influenced by personality traits, mental health, family and educational background, social support, interpersonal relationships, physical activity, healthy lifestyle, psychological state, coping strategies, and socio-cultural and economic factors.  Firstly, personality traits play a significant role in influencing well-being. For example, Wang (2020) emphasized the importance of gratitude in promoting happiness. Research by Li et al. (2021) indicated that neuroticism tends to diminish happiness levels, whereas extraversion generally enhances them. Dong and Ni (2020) demonstrated that emotional regulation plays a crucial role in mediating this relationship. Additionally, Ju et al. (2023) and Su and He (2023) delved into how resilience and belief in a just world can affect happiness. Wu et al. (2022) and Feng and Yang (2024) established connections between self-esteem and achievement motivation and overall well-being. Bi and Li (2021) and Cheng et al. (2021) examined the contributions of psychological flexibility and forgiveness to happiness.  Secondly, both family and educational factors have profound impacts on well-being（Li et al. 2022）. Yang and Wang (2023) revealed that family rituals are closely associated with the mental health of adolescents. Qiu et al. (2023) discovered that labor education can enhance self-efficacy and promote healthy lifestyles among college students. Cheng et al. (2023) and Ran et al. (2023) explored the connections between family dynamics and career exploration, and their relevance to well-being. Xu et al. (2024) also highlighted the influence of social media on happiness, which has important implications for educational contexts.  Subsequently, social support and relationships matter for well-being. Xue et al. (2022) highlighted that engaging in leisure activities can effectively boost happiness. Meanwhile, a series of studies have underscored the positive impact of strong social networks and positive relationships on happiness, including those by Jiang et al. (2021), Wang et al. (2022), Lan and Wang (2024), Zhao (2021), and Huang and Zhang (2022). In addition to social factors, physical exercise and healthy living have also been proven to enhance well-being (Chen et al. 2024). Yuan and You (2022) and Zhang et al. (2022) demonstrated that regular physical activity is beneficial for well-being, while Chen et al. (2021) and Zhang et al. (2021) emphasized the importance of maintaining a healthy diet and sufficient sleep for achieving greater happiness.  Moreover, the way we handle stress has a significant impact on our happiness. Wang et al. (2020) and Hou et al. (2018) investigated how different coping styles shape well-being. Ye et al. (2021) explored how emotional self-efficacy and a sense of life meaning can influence happiness. Jiang et al. (2021) further highlighted the role of emotion regulation in effective coping strategies.  Ultimately, the influence of sociocultural and economic factors on happiness is extensive and multifaceted. For example, Xu et al. (2023) have demonstrated a clear link between career capital and overall well-being, highlighting how economic opportunities and professional development can shape one’s sense of happiness. Liu et al. (2023) have delved into the long-term effects of higher education policies, showing how these policies can have a lasting impact on individuals’ happiness by influencing their educational and career trajectories. Yu et al. (2024) have explored the role of self-determination in goal pursuit, revealing that the cultural emphasis on autonomy and personal goals can significantly affect happiness. Wang and Fu (2024) and Zhang et al. (2024) have also examined how modern lifestyle factors such as internet use and sports anxiety can influence happiness, reflecting the broader sociocultural and economic contexts that shape our daily experiences and well-being. | 6 |
| Risk of bias in studies | 18 | Two authors independently screened studies based on inclusion criteria and discussed and confirmed the risk of bias. | 6 |
| Results of individual studies | 19 | **Table3** Summary of included studies   \| **ID** \| **Author (Year)** \| **Aim of Study** \| **Design** \| **Participants (N)** \| **Primary Variables** \| \| --- \| --- \| --- \| --- \| --- \| --- \| \| 1 \| Cheng et al. (2021) \| Love forgiveness and SWB \| Cross-sectional \| 831 \| Love Forgiveness, Interpersonal Relationships \| \| 2 \| Li et al. (2022) \| Family culture, personality, and SWB \| Cross-sectional \| 340 \| Neuroticism, Extraversion, Family culture \| \| 3 \| Yang & Wang (2023) \| Family rituals and mental health \| Cross-sectional \| 424 \| Family rituals, Cohesion, Adaptability \| \| 4 \| Xue et al. (2022) \| Leisure crafting and well-being \| Diary study \| 80 \| Leisure resources, Social leisure \| \| 5 \| Yuan & You (2022) \| Physical activity during pandemic \| Cross-sectional \| 1,198 \| Physical Activity, Adverse mental health \| \| 6 \| Qiu et al. (2023) \| Labor education and SWB \| Cross-sectional \| 2,028 \| Labor education, Self-efficacy, Lifestyle \| \| 7 \| Cheng et al. (2023) \| Pandemic policies and behavior \| Longitudinal \| 1,641 \| Love forgiveness, Interpersonal relationships \| \| 8 \| Zhang et al. (2021) \| Physical activity and perceived health \| Cross-sectional \| 1,204 \| MVPA guideline, Perceived health \| \| 9 \| Dong & Ni (2020) \| Dispositional awe and SWB \| Cross-sectional \| 332 \| Awe, Openness, Extraversion \| \| 10 \| Ju et al. (2023) \| Strength-based parenting and growth \| Cross-sectional \| 621 \| Parenting, Personal growth, Strengths use \| \| 11 \| Su & He (2023) \| Sleep quality and resilience \| Cross-sectional \| 3,349 \| Sleep quality, Resilience, Just world belief \| \| 12 \| Wu et al. (2022) \| Child maltreatment and self-esteem \| Cross-sectional \| 358 \| Maltreatment, Self-esteem, Self-compassion \| \| 13 \| Feng & Yang (2024) \| Achievement motivation and control \| Cross-sectional \| 1,017 \| Motivation, Self-control, Self-management \| \| 14 \| Zuo et al. (2024) \| Character strengths and social support \| Cross-sectional \| 336 \| Character strengths, Support, Emotions \| \| 15 \| Xu et al. (2024) \| Social media and experiential happiness \| Qualitative \| 5 \| Social media use, Identity, Comparison \| \| 16 \| Jiang et al. (2021) \| Coping styles and emotion regulation \| Cross-sectional \| 1,127 \| Coping styles, Emotion regulation \| \| 17 \| Wang et al. (2022) \| Ambivalence over emotional expression \| Cross-sectional \| 555 \| Fear of intimacy, Attachment avoidance \| \| 18 \| Chen et al. (2024) \| Exercise adherence and character \| Cross-sectional \| 1,001 \| Exercise adherence, Mental character \| \| 19 \| Wang et al. (2018) \| SNS usage and psychological well-being \| Longitudinal \| 265 \| Passive Social Networking Site Usage \| \| 20 \| Huang & Zhang (2022) \| Social support in online learning \| Cross-sectional \| 515 \| Social support, Psychological capital \| \| 21 \| Zhang et al. (2022) \| Physical activity intensity \| Cross-sectional \| 723 \| Vigorous/Moderate physical activity \| \| 22 \| Wang (2020) \| Trait gratitude and basic needs \| Cross-sectional \| 481 \| Trait gratitude, Psychological needs \| \| 23 \| Hou et al. (2018) \| Religiosity and meaningfulness \| Mixed (CS & Qual) \| 1,418 \| Religiosity, Meaningfulness \| \| 24 \| Ran et al. (2023) \| Career adjustment and calling \| Longitudinal \| 1,077 \| Career Exploration/Calling, Self-reflection \| \| 25 \| Xu et al. (2023) \| Career capital effect on well-being \| Cross-sectional \| 312 \| Career capital, Career adaptability \| \| 26 \| Yu et al. (2024) \| Longitudinal trajectories of SWB \| Longitudinal \| 1,050 \| Self-determination, Resilience factors \| \| 27 \| Zhang et al. (2024) \| Sport anxiety and need satisfaction \| Cross-sectional \| 835 \| Sport anxiety, Need satisfaction \| \| 28 \| Wang & Fu (2024) \| Internet addiction and social support \| Cross-sectional \| 681 \| Internet addiction, Meaning in life \| \| 29 \| Lan & Wang (2024) \| Socio-economic status and values \| Cross-sectional \| 600 \| Values, Socio-economic status \| \| 30 \| Liu et al. (2023) \| Education expansion and SWB \| Quasi-exp \| 15,301 \| Higher Education Expansion policy \| \| 31 \| Liu et al. (2019) \| Authoritarian personality \| Cross-sectional \| 1,007 \| Authoritarianism, Organizational culture \| \| 32 \| Zhao (2021) \| Social media addiction \| Cross-sectional \| 3,370 \| Social media use/addiction \| \| 33 \| Ye et al. (2021) \| Social anxiety and emotional efficacy \| Cross-sectional \| 908 \| Social anxiety, Emotional self-efficacy \| \| 34 \| Bi & Li (2021) \| Psychological flexibility profiles \| Cross-sectional \| 644 \| Psychological flexibility, Adjustment \| | 12 |
| Results of syntheses | 20a | All included studies are empirical research written in English, focusing on Chinese college students and published between 2018 and 2024. The primary variable across all studies is college students' subjective well-being, with each investigation examining factors influencing changes in this well-being. | 18 |
|  | 20b | The articles included in the study all describe factors influencing changes in subjective well-being and provide empirical data demonstrating that these factors are statistically significant. | 10 |
|  | 20c | Research on non-English-language studies targeting Chinese college students, and subjective well-being is not a primary variable. | 10 |
|  | 20d | Compare the results of different types of studies (randomized controlled trials versus observational studies). | 10 |
| Reporting biases | 21 | Data from the Chinese database was omitted. | 17 |
| Certainty of evidence | 22 | Data certainty was confirmed through the independent work of two authors and subsequent discussion. | 10 |
| **DISCUSSION** | | |  |
| Discussion | 23a | This study findings are in alignment with the principles of self-determination theory(Ryan & Deci, 2017). This correspondence provides substantial empirical evidence in support of the application of the theory among college students. | 21 |
|  | 23b | The data from the Chinese database is missing, and no meta-analysis was conducted. | 17 |
|  | 23c | No quantitative or qualitative research was conducted to establish distinct screening criteria; only whether the study was empirical was considered. | 10 |
|  | 23d | The factors influencing the changes in Chinese college students' subjective well-being before and during the COVID-19 pandemic are multifaceted, including personality traits and mental health, family and educational background, social support and interpersonal relationships, physical activity and healthy lifestyle, as well as psychological state and coping strategies. Addressing these factors is crucial for students' holistic development and success. Educational institutions can play a pivotal role by implementing comprehensive strategies and fostering supportive environments to safeguard students' mental health and well-being. The lessons learned during the pandemic can inform the development of more resilient and adaptive educational frameworks, ultimately contributing to a healthier and more supportive learning environment for future generations. | 18 |
| **OTHER INFORMATION** | | |  |
| Registration and protocol | 24a | Not registered | 18 |
|  | 24b | No plan has been formulated | 18 |
|  | 24c | Not applicable | 18 |
| Support | 25 | Not applicable | 18 |
| Competing interests | 26 | There is no conflict of interest between the authors of this article. | 18 |
| Availability of data, code and other materials | 27 | Across the PubMed, Web of Science, and Scopus databases. | 18 |

*From:*  Page MJ, McKenzie JE, Bossuyt PM, Boutron I, Hoffmann TC, Mulrow CD, et al. The PRISMA 2020 statement: an updated guideline for reporting systematic reviews. BMJ 2021;372:n71. doi: 10.1136/bmj.n71. This work is licensed under CC BY 4.0. To view a copy of this license, visit <https://creativecommons.org/licenses/by/4.0/>
