# Supplementary material for: A systematic review of predictors of college students’ subjective well-being: evidence from pre- and post-pandemic literature
Source: Front Public Health. 2026 Apr 16;14:1793063. doi: 10.3389/fpubh.2026.1793063 (PMC13128366; doi:10.3389/fpubh.2026.1793063)
Supplement: Supplementary file 3 [file Table_3.docx]

1.JBI Critical Appraisal Checklist for
analytical cross sectional studies

Reviewer ______________________________________ Date_______________________________

Author_______________________________________ Year_________ Record Number_________

|  | **Yes** | **No** | **Unclear** | **Not applicable** |
| --- | --- | --- | --- | --- |
| 1. Were the criteria for inclusion in the sample clearly defined? | □ | □ | □ | □ |
| 1. Were the study subjects and the setting described in detail? | □ | □ | □ | □ |
| 1. Was the exposure measured in a valid and reliable way? | □ | □ | □ | □ |
| 1. Were objective, standard criteria used for measurement of the condition? | □ | □ | □ | □ |
| 1. Were confounding factors identified? | □ | □ | □ | □ |
| 1. Were strategies to deal with confounding factors stated? | □ | □ | □ | □ |
| 1. Were the outcomes measured in a valid and reliable way? | □ | □ | □ | □ |
| 1. Was appropriate statistical analysis used? | □ | □ | □ | □ |

Overall appraisal: Include □ Exclude □ Seek further info □

Comments (Including reason for exclusion)

________________________________________________________________________________________________________________________________________________________________________________________________

2.JBI Critical Appraisal Checklist for
Qualitative Research

Reviewer ______________________________________ Date_______________________________

Author_______________________________________ Year_________ Record Number_________

|  | **Yes** | **No** | **Unclear** | **Not applicable** |
| --- | --- | --- | --- | --- |
| 1. Is there congruity between the stated philosophical perspective and the research methodology? | □ | □ | □ | □ |
| 1. Is there congruity between the research methodology and the research question or objectives? | □ | □ | □ | □ |
| 1. Is there congruity between the research methodology and the methods used to collect data? | □ | □ | □ | □ |
| 1. Is there congruity between the research methodology and the representation and analysis of data? | □ | □ | □ | □ |
| 1. Is there congruity between the research methodology and the interpretation of results? | □ | □ | □ | □ |
| 1. Is there a statement locating the researcher culturally or theoretically? | □ | □ | □ | □ |
| 1. Is the influence of the researcher on the research, and vice- versa, addressed? | □ | □ | □ | □ |
| 1. Are participants, and their voices, adequately represented? | □ | □ | □ | □ |
| 1. Is the research ethical according to current criteria or, for recent studies, and is there evidence of ethical approval by an appropriate body? | □ | □ | □ | □ |
| 1. Do the conclusions drawn in the research report flow from the analysis, or interpretation, of the data? | □ | □ | □ | □ |

Overall appraisal: Include □ Exclude □ Seek further info □

Comments (Including reason for exclusion

________________________________________________________________________________________________________________________________________________________________________________________________

3.JBI Critical Appraisal Checklist for cohort studies

Reviewer ______________________________________ Date_______________________________

Author_______________________________________ Year_________ Record Number_________

|  | **Yes** | **No** | **Unclear** | **Not applicable** |
| --- | --- | --- | --- | --- |
| 1. Were the two groups similar and recruited from the same population? | □ | □ | □ | □ |
| 1. Were the exposures measured similarly to assign people to both exposed and unexposed groups? | □ | □ | □ | □ |
| 1. Was the exposure measured in a valid and reliable way? | □ | □ | □ | □ |
| 1. Were confounding factors identified? | □ | □ | □ | □ |
| 1. Were strategies to deal with confounding factors stated? | □ | □ | □ | □ |
| 1. Were the groups/participants free of the outcome at the start of the study (or at the moment of exposure)? | □ | □ | □ | □ |
| 1. Were the outcomes measured in a valid and reliable way? | □ | □ | □ | □ |
| 1. Was the follow up time reported and sufficient to be long enough for outcomes to occur? | □ | □ | □ | □ |
| 1. Was follow up complete, and if not, were the reasons to loss to follow up described and explored? | □ | □ | □ | □ |
| 1. Were strategies to address incomplete follow up utilized? | □ | □ | □ | □ |
| 1. Was appropriate statistical analysis used? | □ | □ | □ | □ |

Overall appraisal: Include □ Exclude □ Seek further info □

Comments (Including reason for exclusion)

________________________________________________________________________________________________________________________________________________________________________________________________

1. JBI checklist for quasi-experimental

| **RoB Assessor:** | | **Date of Appraisal:** | | **Record Number:** | | | | |
| --- | --- | --- | --- | --- | --- | --- | --- | --- |
| **Study Author:** | | **Study Title:** | | **Study Year:** | | | | |
|  | |  | |  | | | | |
| **Internal Validity** | | | **Choice - Comments/Justification** | | **Yes** | **No** | **Unclear** | **N/A** |
| **Bias related to temporal precedence** | | | | | | | | |
| **1** | **Is it clear in the study what is the “cause” and what is the “effect” (i.e. there is no confusion about which variable comes first)?** | |  | |  |  |  |  |
| **Bias related to selection and allocation** | | | | | | | | |
| **2** | **Was there a control group?** | |  | |  |  |  |  |
| **Bias related to confounding factors** | | | | | | | | |
| **3** | **Were participants included in any comparisons similar?** | |  | |  |  |  |  |
| **Bias related to administration of intervention/exposure** | | | | | | | | |
| **4** | **Were the participants included in any comparisons receiving similar treatment/care, other than the exposure or intervention of interest?** | |  | |  |  |  |  |

| **Bias related to assessment, detection and measurement of the outcome** | | | | | | |
| --- | --- | --- | --- | --- | --- | --- |
| **5** | **Were there multiple measurements of the outcome, both pre and post the intervention/exposure?** |  | **Yes** | **No** | **Unclear** | **N/A** |
|  | **Outcome 1** |  |  |  |  |  |
|  | **Outcome 2** |  |  |  |  |  |
|  | **Outcome 3** |  |  |  |  |  |
|  | **Outcome 4** |  |  |  |  |  |
|  | **Outcome 5** |  |  |  |  |  |
|  | **Outcome 6** |  |  |  |  |  |
|  | **Outcome 7** |  |  |  |  |  |
|  |  |  |  |  |  |  |
| **6** | **Were the outcomes of participants included in any comparisons measured in the same way?** |  | **Yes** | **No** | **Unclear** | **N/A** |
|  | **Outcome 1** |  |  |  |  |  |
|  | **Outcome 2** |  |  |  |  |  |
|  | **Outcome 3** |  |  |  |  |  |
|  | **Outcome 4** |  |  |  |  |  |
|  | **Outcome 5** |  |  |  |  |  |
|  | **Outcome 6** |  |  |  |  |  |
|  | **Outcome 7** |  |  |  |  |  |
|  |  |  |  |  |  |  |
| **7** | **Were outcomes measured in a reliable way?** |  | **Yes** | **No** | **Unclear** | **N/A** |
|  | **Outcome 1** |  |  |  |  |  |
|  | **Outcome 2** |  |  |  |  |  |
|  | **Outcome 3** |  |  |  |  |  |
|  | **Outcome 4** |  |  |  |  |  |
|  | **Outcome 5** |  |  |  |  |  |
|  | **Outcome 6** |  |  |  |  |  |
|  | **Outcome 7** |  |  |  |  |  |

| **Bias related to participant retention** | | | | | | | | | | | | | | | |
| --- | --- | --- | --- | --- | --- | --- | --- | --- | --- | --- | --- | --- | --- | --- | --- |
| **8** | **Was follow-up complete and if not, were differences between groups in terms of their follow-up adequately described and analyzed?** | | | | | |  | |  | | | | | | |
|  | **Outcome 1** | | | | | |  | | **Yes** | **No** | | **Unclear** | | **N/A** | |
|  |  | Result 1 | | | | |  | |  |  |  | | |  | |
|  |  | Result 2 | | | | |  | |  |  |  | | |  | |
|  |  | Result 3 | | | | |  | |  |  |  | | |  | |
|  | **Outcome 2** | | | | | |  | | **Yes** | **No** | **Unclear** | | | **N/A** | |
|  |  | Result 1 | | | | |  | |  |  |  | | |  | |
|  |  | Result 2 | | | | |  | |  |  |  | | |  | |
|  |  | Result 3 | | | | |  | |  |  |  | | |  | |
|  | **Outcome 3** | | | | | |  | | **Yes** | **No** | **Unclear** | | | **N/A** | |
|  |  | Result 1 | | | | |  | |  |  |  | | |  | |
|  |  | Result 2 | | | | |  | |  |  |  | | |  | |
|  |  | Result 3 | | | | |  | |  |  |  | | |  | |
|  | **Outcome 4** | | | | | |  | | **Yes** | **No** | **Unclear** | | | **N/A** | |
|  |  | Result 1 | | | | |  | |  |  |  | | |  | |
|  |  | Result 2 | | | | |  | |  |  |  | | |  | |
|  |  | Result 3 | | | | |  | |  |  |  | | |  | |
|  | **Outcome 5** | | | | | |  | | **Yes** | **No** | **Unclear** | | | **N/A** | |
|  |  | Result 1 | | | | |  | |  |  |  | | |  | |
|  |  | Result 2 | | | | |  | |  |  |  | | |  | |
|  |  | Result 3 | | | | |  | |  |  |  | | |  | |
|  | **Outcome 6** | | | | | |  | | **Yes** | **No** | **Unclear** | | | **N/A** | |
|  |  | Result 1 | | | | |  | |  |  |  | | |  | |
|  |  | Result 2 | | | | |  | |  |  |  | | |  | |
|  |  | Result 3 | | | | |  | |  |  |  | | |  | |
|  | **Outcome 7** | | | | | |  | | **Yes** | **No** | **Unclear** | | | **N/A** | |
|  |  | Result 1 | | | | |  | |  |  |  | | |  | |
|  |  | Result 2 | | | | |  | |  |  |  | | |  | |
|  |  | Result 3 | | | | |  | |  |  |  | | |  | |
|  |  |  | | | | | | |  |  |  | | |  | |
|  | **Statistical Conclusion Validity** | | | | | | | |  |  |  | | |  | |
| **9** | **Was appropriate statistical analysis used?** | | | | | | |  |  | | | | | | |
|  | **Outcome 1** | | |  | | | |  | **Yes** | **No** | **Unclear** | | | **N/A** | |
|  |  | Result 1 | | | | | |  |  |  |  | | |  | |
|  |  | Result 2 | | | | | |  |  |  |  | | |  | |
|  |  | Result 3 | | | | | |  |  |  |  | | |  | |
|  | **Outcome 2** | | |  | | | |  | **Yes** | **No** | **Unclear** | | | **N/A** | |
|  |  | Result 1 | | | | | |  |  |  |  | | |  | |
|  |  | Result 2 | | | | | |  |  |  |  | | |  | |
|  |  | Result 3 | | | | | |  |  |  |  | | |  | |
|  | **Outcome 3** | | |  | | | |  | **Yes** | **No** | **Unclear** | | | **N/A** | |
|  |  | Result 1 | | | | | |  |  |  |  | | |  | |
|  |  | Result 2 | | | | | |  |  |  |  | | |  | |
|  |  | Result 3 | | | | | |  |  |  | | |  | |  |
|  | **Outcome 4** | | | | | | |  | **Yes** | **No** | | | **Unclear** | | **N/A** |
|  |  | Result 1 | | | | | |  |  |  | | |  | |  |
|  |  | Result 2 | | | | | |  |  |  | | |  | |  |
|  |  | Result 3 | | | | | |  |  |  | | |  | |  |
|  | **Outcome 5** | | | | | | |  | **Yes** | **No** | | | **Unclear** | | **N/A** |
|  |  | Result 1 | | | | | |  |  |  | | |  | |  |
|  |  | Result 2 | | | | | |  |  |  | | |  | |  |
|  |  | Result 3 | | | | | |  |  |  | | |  | |  |
|  | **Outcome 6** | | | | | | |  | **Yes** | **No** | | | **Unclear** | | **N/A** |
|  |  | Result 1 | | | | | |  |  |  | | |  | |  |
|  |  | Result 2 | | | | | |  |  |  | | |  | |  |
|  |  | Result 3 | | | | | |  |  |  | | |  | |  |
|  | **Outcome 7** | | | | | | |  | **Yes** | **No** | | | **Unclear** | | **N/A** |
|  |  | Result 1 | | | | | |  |  |  | | |  | |  |
|  |  | Result 2 | | | | | |  |  |  | | |  | |  |
|  |  | Result 3 | | | | | |  |  |  | | |  | |  |
|  | | | | | | | | | | | | | | | |
| **Overall appraisal:** | | | **Include:** | | **Exclude:** | **Seek Further Info:** | | | | | | | | | |
| **Comments:** | | | | | | | | | | | | | | | |
